# Editorial: Biomedical signals and artificial intelligence towards smart robots control strategies

**DOI:** 10.3389/fnbot.2025.1570870

**Published:** 2025-04-25

**Authors:** Hassene Seddik, Hassen Fourati, Chiraz Ben Jabeur, Nahla Khraief

**Affiliations:** ^1^Department of Computer and Embedded Systems, Higher National Engineering School of Tunis, Tunis, Tunisia; ^2^Department of Automatic Control, Université Grenoble Alpes, Saint Martin d'Héres, Auvergne-Rhone-Alpes, France; ^3^Department of Information Systems, Higher Institute of Informatics (ISI), Ariana, Tunisia; ^4^Department of Electrical Engineering, National Engineering School of Tunis (ENIT), Tunis, Tunisia

**Keywords:** artificial intelligence, robotics, control, trajectory planning, biomedical signal analysis

## 1 Introduction

Robotic systems have significantly evolved, integrating Artificial Intelligence (AI) techniques to enhance autonomy, adaptability, and robustness. Traditional control methods, such as Proportional-Integral-Derivative (PID) controllers, remain fundamental in many applications, yet AI-driven approaches are proving invaluable in tackling complex, non-linear, and uncertain environments.

This Research Topic explores the latest advancements in AI-driven control techniques for robotic applications. The contributions cover a range of topics, including adaptive and learning-based control strategies, intelligent navigation, state estimation, and the integration of hybrid AI techniques for enhanced robotic performance.

## 2 Overview of contributions

The articles included in this Research Topic address various challenges in robotic control and introduce innovative AI-based methodologies. Below, we highlight the key contributions:

- **Intelligent control techniques:** Several articles explore the integration of AI-driven controllers, such as neural networks and fuzzy logic, to improve robotic decision-making and adaptability in dynamic environments.
- **State estimation and sensor fusion:** Advanced techniques like Kalman filters and particle filters are leveraged to enhance real-time localization and trajectory tracking, ensuring precise motion planning.
- **Hybrid control strategies:** The fusion of classical and AI-based methods demonstrates how hybrid controllers can achieve robust performance, particularly in unstructured and uncertain scenarios.
- **Obstacle avoidance and path optimization:** Contributions discuss AI-enhanced navigation algorithms that enable robots to autonomously detect obstacles and compute optimal paths in real time.

## 3 Summary of accepted articles in the Research Topic

The first article, “*On designing a configurable UAV autopilot for unmanned quadrotors*,” presents a control strategy for unmanned aerial vehicles (UAVs) based on an inner-outer loop design for attitude and altitude control (Bhar and Sayadi). It leverages nonlinear feedback linearization, linear-quadratic regulator (LQR), sliding mode control (SMC), proportional-derivative (PD), and PID controllers to enhance stability and disturbance rejection. The study also introduces a PD–PID hybrid controller designed for tracking and surveillance of smoke and fire. Simulation results confirm the effectiveness of the proposed approach in improving UAV performance under various conditions.

The second article, “*Fusion inception and transformer network for continuous estimation of finger kinematics from surface electromyography (sEMG)*,” introduces FIT (Fusion Inception Transformer), a deep learning model for decoding sEMG signals to predict hand movements (Lin and Zhang). By integrating Inception and Transformer networks, the model efficiently extracts both local and global features. Results on the Ninapro dataset show that FIT outperforms temporal convolutional networks (TCN), long short-term memory (LSTM) networks, and Bidirectional Encoder Representations from Transformers (BERT) in terms of estimation accuracy and computational efficiency, contributing significantly to advancements in human-computer interaction (HCI) technology.

The third article, *"Cardioid oscillator-based pattern generator for imitating lower limb exoskeleton behavior,"* proposes a pattern generator based on cardioid oscillators to mimic human gait characteristics such as periodicity, self-excitation, and time-ratio asymmetry (Fu et al.). The approach is validated through simulations and experiments, demonstrating that the generated trajectories closely resemble natural human gait. This method holds promise for exoskeleton control in rehabilitation and assistive applications.

The fourth article, *"An autonomous mobile robot path planning strategy using an enhanced slime mold algorithm (ESMA),"* introduces ESMA, an improved optimization method for robotic path planning (Zheng et al.). By incorporating adaptive techniques for enhanced global search and an artificial potential field for dynamic obstacle avoidance, ESMA significantly reduces both path length and computation time. Comparative analysis against the standard Slime Mold Algorithm (SMA) and other optimization algorithms confirms its superiority, making it a valuable solution for real-world robotic navigation.

The fifth article, *"Multi-user motion recognition using sEMG via discriminative canonical correlation analysis and adaptive dimensionality reduction,"* proposes a multi-user sEMG motion recognition framework designed to address variability in muscle signals across different users (Wang et al.). By utilizing discriminative canonical correlation analysis (DCCA) and adaptive dimensionality reduction (ADR), the system projects feature sets into a uniform space, enhancing motion recognition accuracy. Experimental results demonstrate over 90% accuracy, highlighting its potential for rehabilitation and assistive technology applications.

## 4 Future perspectives

The convergence of AI and robotics presents exciting research opportunities. Future advancements may focus on reinforcement learning for real-time adaptation, neuromorphic computing for energy-efficient robotic control, and explainable AI (XAI) for transparent decision-making in safety-critical applications.

## 5 Conclusion

This Research Topic highlights the growing role of AI in robotic applications and control. The featured articles provide valuable insights into emerging methodologies that push the boundaries of autonomous robotic systems. We thank all the contributors for their efforts in advancing this field.

